# Influence of Low Loadings of Cellulose Nanocrystals on the Simultaneously Enhanced Crystallization Rate, Mechanical Property, and Hydrophilicity of Biobased Poly(butylene 2,5-furandicarboxylate)

**DOI:** 10.3390/polym17020196

**Published:** 2025-01-14

**Authors:** Siyu Pan, Haidong Yang, Zhaobin Qiu

**Affiliations:** State Key Laboratory of Chemical Resource Engineering, Beijing University of Chemical Technology, Beijing 100029, China; 2022400087@grad.buct.edu.cn (S.P.); 2023200346@grad.buct.edu.cn (H.Y.)

**Keywords:** biobased, CNC, composites

## Abstract

In this research, fully biobased composites consisting of poly(butylene 2,5-furandicarboxylate) (PBF) and cellulose nanocrystals (CNC) were successfully prepared through a common solution and casting method. The influence of CNC on the crystallization behavior, mechanical property, and hydrophilicity of PBF was systematically investigated. Under different crystallization processes, the crystallization of PBF was obviously promoted by CNC as a biobased nucleating agent. The Ozawa equation was not suitable to fit the nonisothermal melt crystallization kinetics of PBF and PBF/CNC composites. The nucleation activity of CNC was quantitatively calculated by the Dobreva method; moreover, the nucleation efficiency of CNC was further evaluated through the self-nucleation procedure. The isothermal melt crystallization kinetics of PBF and PBF/CNC composites was well described by the Avrami method; moreover, the crystallization mechanism and the crystal structure of PBF remained unchanged despite the presence of CNC. CNC also greatly enhanced both the mechanical property and hydrophilicity of PBF in the composites. In sum, low loadings of CNC simultaneously improved the crystallization, mechanical property, and hydrophilicity of PBF, which should be of significant importance and interest in fully biobased polymer composites from a sustainable viewpoint.

## 1. Introduction

As a biobased rigid monomer, 2,5-furandicarboxylic acid (FDCA) has recently attracted widespread research interests in both academic and industrial fields. FDCA has been selected by the US Department of Energy as one of the top value-added chemicals [1,2,3,4,5]. In the literature, many FDCA-based polyesters have already been synthesized and studied, including poly(ethylene 2,5-furandicarboxylate) (PEF), poly(hexamethylene 2,5-furandicarboxylate) (PHF), and poly(butylene 2,5-furandicarboxylate) (PBF) [6,7,8,9,10]. Among them, PBF, with good mechanical property, outstanding gas barrier property, and high thermal stability, is expected to replace fossil-based poly(butylene terephthalate) (PBT) in some industrial fields [11,12,13,14]. Although the chemical structure, thermal behavior, and mechanical property of PBF are comparable to those of PBT, PBF actually shows a much slower crystallization rate than PBT due to the asymmetric furan ring structure [15,16]. From the viewpoint of polymer processing, it is essential to accelerate the crystallization of PBF to promote its industrial applications. Some PBF-based composites have recently been prepared and investigated to adjust the physical properties of PBF [17,18,19,20,21,22]. For instance, Wang et al. fabricated PBF/talc composites by a melt-blending process [17]. They reported that talc platelets not only promoted the nonisothermal crystallization of PBF by simultaneously reducing crystallization activation energy and increasing nucleation activity but also enhanced the mechanical properties of PBF. Bikiaris et al. synthesized three types of montmorillonites-modified PBF nanocomposites through an in situ two-step polycondensation and reported that the nanofillers remarkably promoted the crystallization of PBF as nucleation agents, as evidenced by the enhanced cold crystallization behavior and smaller crystallization half-time [19,20].

Cellulose nanocrystals (CNCs) show unique rod-like morphology, low density, and high crystallinity as a biobased nanofiller [23,24,25]. Due to the large specific surface area and relatively high aspect ratio, CNC may behave as an efficient nucleation agent and promote the crystallization of some polyesters [26,27]. In addition, CNC can also enhance both the hydrophilicity and mechanical properties of polymer matrix due to the abundant hydroxyl groups on its surface and its high mechanical strength [27,28,29]. In previous studies, our lab once fabricated and studied some biobased and/or biodegradable polyester/CNC composites [30,31,32,33,34,35,36,37,38,39]. In these composites, CNC both nucleated and reinforced the polymer matrix to some degree; moreover, the advantage of CNC did not deteriorate the biobased and/or biodegradable feature of polymer matrix, which should be of great importance from a sustainable viewpoint [30,31,32,33,34,35,36,37,38,39].

In this work, we prepared fully biobased composites consisting of PBF and low loadings of CNC (0.5 and 1 wt%) and studied the influence of CNC on the physical properties of PBF in detail. The novelties of this research were as follows. Firstly, we prepared and reported fully biobased PBF/CNC composites. Secondly, CNC greatly improved the physical properties of PBF, including crystallization, mechanical property, and hydrophilicity, thereby promoting the practical application of PBF from a sustainable perspective. The research results are expected to be significantly interesting and important in fully biobased polymer composites.

## 2. Experimental Section

### 2.1. Materials

The PBF used in this work was synthesized via a two-stage transesterification and polycondensation reaction in our laboratory [40]. The intrinsic viscosity ([*η*]) of PBF was 0.81 dL/g, which was measured with an Ubbelohde viscometer in a mixed solvent of 1,1,2,2-tetrachloroethane and phenol (*w*/*w*, 1:1) at 25 °C. Cellulose nanocrystals were bought from Shanghai ScienceK Nanotechnology Co., Ltd. (Shanghai, China). According to the supplier, the average diameter of CNC was about 5~20 nm, while the length was about 50~200 nm.

### 2.2. Preparation of PBF/CNC Composites

Through a common solution and casting process, two PBF/CNC composites were prepared. The composite consisting of 0.5 wt% of CNC was abbreviated as PBF/CNC0.5, while the other was named as PBF/CNC1. The reason why only a limited range of CNC loadings was used may be explained as follows. In this work, we used unmodified CNC, which was hydrophilic, while PBF was hydrophobic. Due to the abundant hydroxyl groups on the surface of CNC, a high content of CNC would lead to severe self-agglomeration, which would further affect the physical properties of PBF. Although low loadings of CNC were used, the crystallization, mechanical properties, and hydrophilicity of PBF were simultaneously improved.

Taking PBF/CNC1 as an example, the preparation procedure was briefly described. At first, 1.98 g of PBF was added into 50 mL of 1,1,1,3,3,3-Hexafluoro-2-propanol/chloroform (10/90, *w*/*w*) mixed solution, and the solution was stirred for 2 h at 38 °C. Meanwhile, 20 mg of CNC was dispersed into 10 mL of chloroform with the help of the sonication at 350 W for 2 h (KQ-700DE ultrasonic generator, Kunshan, China) to form a suspension. Later, CNC suspension and PBF solution were further mixed and stirred for 4 h at 38 °C and then cast into a dish. Third, the solvent was evaporated to form a film at room temperature in a hood. Finally, the film was dried at 40 °C for 120 h in a vacuum oven to remove the residual solvent completely.

**Scheme 1:**
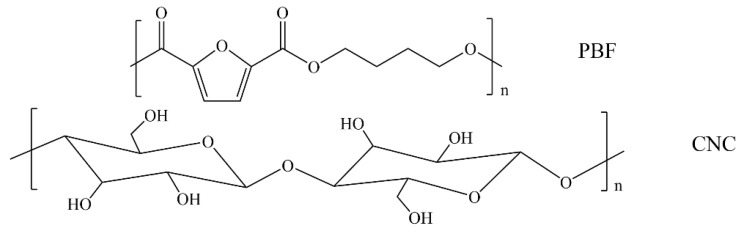
Chemical structures of PBF and CNC.

Scheme 1 illustrates the chemical structures of PBF and CNC as follows.

### 2.3. Characterizations

The crystallization behavior of PBF and PBF/CNC composites was investigated using a TA Q100 differential scanning calorimeter (DSC) (TA Instruments Q100, New Castle, DE, USA) under a nitrogen atmosphere. Before each measurement, any previous thermal history of the sample was eliminated by annealing at 210 °C for 3 min. For the nonisothermal melt crystallization study, the sample was cooled to −25 °C at different cooling rates from 5 to 20 °C/min. While in the case of isothermal melt crystallization kinetics study, the sample was rapidly cooled to the selected crystallization temperature (*T*_c_ = 145−155 °C) at 60 °C/min and kept at *T*_c_ for a period of time to ensure complete crystallization.

The self-nucleation of PBF was also investigated with DSC. The specific DSC temperature-controlled procedures, which are clearly described in Figure 4a in the following section, include the following 7 steps:

In steps 1 to 3, a standard crystallization procedure was established, while in steps 4 to 6, a self-nucleation procedure was completed. Finally, in step 7, the melting behavior was studied to judge the occurrence of self-nucleation and annealing. In step 1, the annealing temperature was set as 210 °C, while in step 4, a lower indicated self-nucleation temperature (*T*_s_) was chosen. The heating rate in step 1 was 40 °C/min, while the cooling and heating rates in other steps were 10 °C/min.

The crystal structures of PBF and PBF/CNC composites were analyzed on a Rigaku Ultima IV X-ray diffractometer over a 2θ range of 5° to 45° with a scanning rate of 5°/min.

The tensile properties of all samples were studied on a universal tensile testing machine (UTM5205XHD) according to the ASTM D638 standard. The dumbbell-shaped films were prepared by hot pressing with a length of 50 mm, a width of 4 mm, and a thickness of 1.0 mm. The tensile rate was set at 20 mm/min, and each sample was tested at least three times.

The water contact angles (WCA) of all samples were measured with an optical contact angle measuring instrument (OCA 50AF) at 24 VDC line voltage and 70 W input power.

## 3. Results and Discussion

### 3.1. Nonisothermal Melt Crystallization Behavior Study

**Figure 1:**
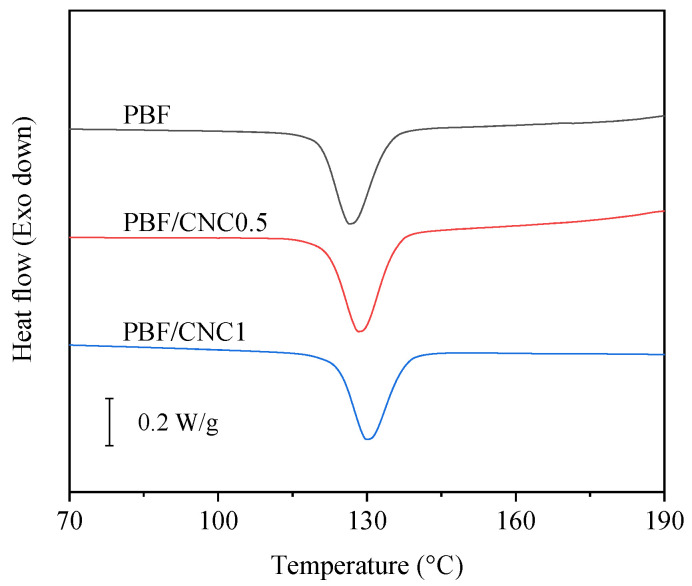
Melt crystallization exotherms of PBF and PBF/CNC composites at 5 °C/min.

The effect of CNC on the enhanced crystallization behavior of PBF was first investigated. The nonisothermal melt crystallization behavior of PBF and PBF/CNC composites was studied with DSC at different cooling rates. Figure 1 displays the crystallization exotherms of PBF and PBF/CNC composites from the crystal-free molten state at a cooling rate of 5 °C/min after erasing previous thermal history as an example. As clearly illustrated in Figure 1, PBF showed a melt crystallization temperature (*T*_p_) of 126.6 °C, while those of PBF/CNC0.5 and PBF/CNC1 gradually increased to 128.5 and 130.0 °C, respectively. In addition, the melt crystallization enthalpy (Δ*H*_p_) of PBF increased from 48.8 J/g to 51.1 and 51.4 J/g after the incorporation of 0.5 and 1 wt% of CNC, respectively, in the composites. The increase in both *T*_p_ and Δ*H*_p_ revealed that the nonisothermal melt crystallization of PBF was enhanced to some degree by CNC as a biobased nucleating agent. In our previous study, the *T*_p_ and Δ*H*_p_ values increased from 105.1 °C and 51.9 J/g for PHF to 112.5 °C and 54.4 J/g for PHF/CNC1, respectively [32]. Obviously, under the same crystallization condition, the improvement of CNC on the crystallization of PHF was stronger than that of PBF. In addition, PPF could not crystallize in the presence of 0.5 wt% of CNC during the cooling process even at 2.5 °C/min, indicating that the crystallization promotion effect of CNC in PBF was better than that of PPF [30]. Figure S1 shows the DSC results of PBF and PBF/CNC composites at different cooling rates ranging from 5 to 20 °C/min. For each sample, crystallization exotherms shifted to a low-temperature range with an increasing cooling rate. Such a result was reasonable in polymer crystallization, as the sample did not have sufficient time to crystallize at a high temperature during a fast cooling process. Table S1 lists the *T*_p_ and Δ*H*_p_ values for PBF and PBF/CNC composites after crystallizing at different cooling rates. With a decreasing cooling rate (*Φ*) or increasing CNC loading, the *T*_p_ and Δ*H*_p_ values gradually increased, suggesting that the nonisothermal melt crystallization of PBF was accordingly promoted.

**Figure 2:**
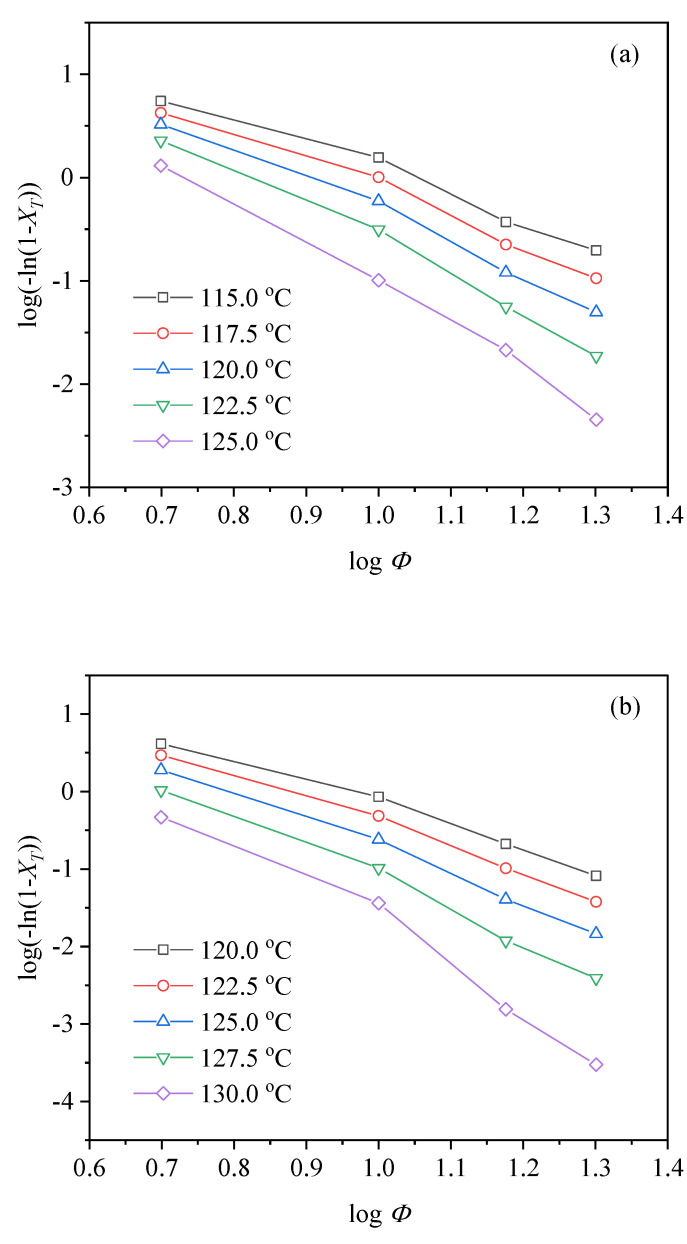

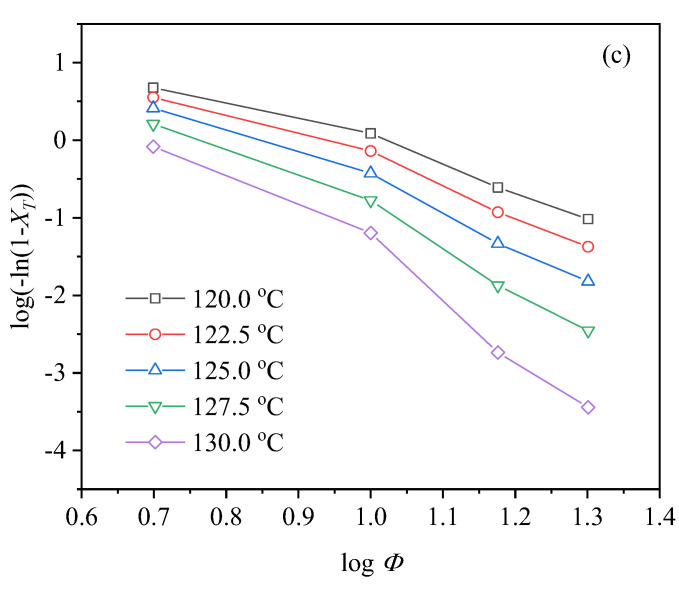
Ozawa plots of (**a**) PBF, (**b**) PBF/CNC0.5, and (**c**) PBF/CNC1.

In the literature, the Ozawa equation is a valid and classical method to study the nonisothermal melt crystallization kinetics of semicrystalline polymers [41,42]. According to the Ozawa equation, the relative crystallinity (*X_T_*) shows the following relationship with the cooling rate and cooling function *K*(*T*) at crystallization temperature (*T*).

(1) $$X_{T}=1-\exp(\frac{-K(T)}{\Phi^{m}})$$

where *m* is the Ozawa exponent, relating to the nucleation and growth mechanism [41,42]. If the nonisothermal melt crystallization kinetics of semicrystalline polymers can be successfully described by the Ozawa method, a series of straight-fitting lines should be acquired [41,42]. Figure 2 depicts the Ozawa plots of PBF and PBF/CNC composites at indicated temperatures. As illustrated in Figure 2, five nonlinear curves were observed for both PBF and PBF/CNC composites. As a result, the Ozawa equation was not suitable to study the nonisothermal melt crystallization of PBF and PBF/CNC composites, which was probably due to the occurrence of the secondary crystallization [42]. A similar result was reported in poly(ethylene succinate) (PES)/CNC composites [33].

**Figure 3:**
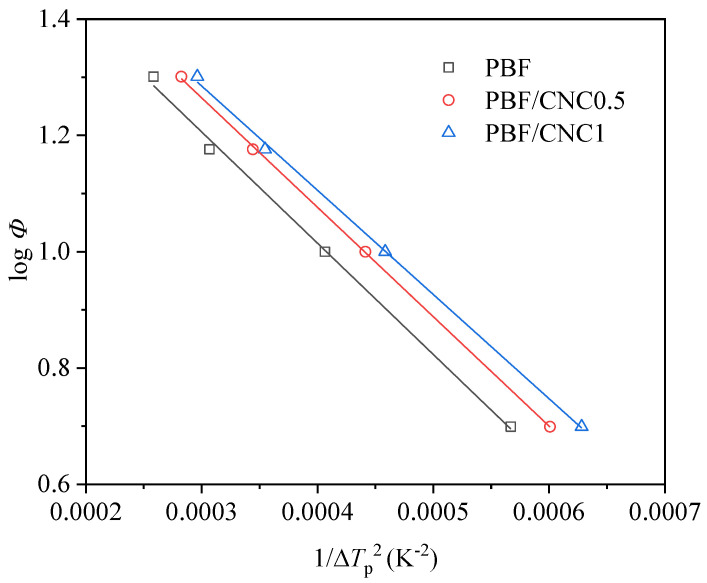
Plots of log *Φ* versus 1/∆*T*_p_^2^ for calculating the nucleation activity.

Through the following two equations proposed by Dobreva et al. [43,44], the nucleation activity (*N*_a_) of CNC was quantitatively studied in PBF/CNC composites:

(2) log Φ=A−B2.303ΔTp2

(3) log Φ=A−B∗2.303ΔTp2

where ∆*T*_p_ is the degree of supercooling, *Φ* is the cooling rate, and *A*, *B*, and B* are the constants. *N*_a_ can be derived from the ratio of B*/*B*; furthermore, the nucleation activity of the foreign substrate is inversely proportional to the value of *N*_a_. From Figure 3, the *N*_a_ values were determined to be 0.98 for PBF/CNC0.5 and 0.94 for PBF/CNC1, respectively. The *N*_a_ values were 0.43 and 0.58 for PES/CNC0.5 and PES/CNC1, respectively, revealing that the nucleation activity of CNC is stronger in flexible aliphatic polyesters than in rigid aromatic polyesters [33].

The nucleation efficiency (*NE*) of CNC was further investigated through the following equation:

(4) $$NE(\%)=\frac{T_{cn}-T_{cc}}{T_{cs}-T_{cc}}\times 100\%$$

where *T*_cc_ is the melt crystallization temperature of PBF while *T*_cn_ is that of PBF/CNC composites at 10 °C/min, and *T*_cs_ is the maximum melt crystallization temperature of the self-nucleated PBF matrix [45,46,47].

**Figure 4:**
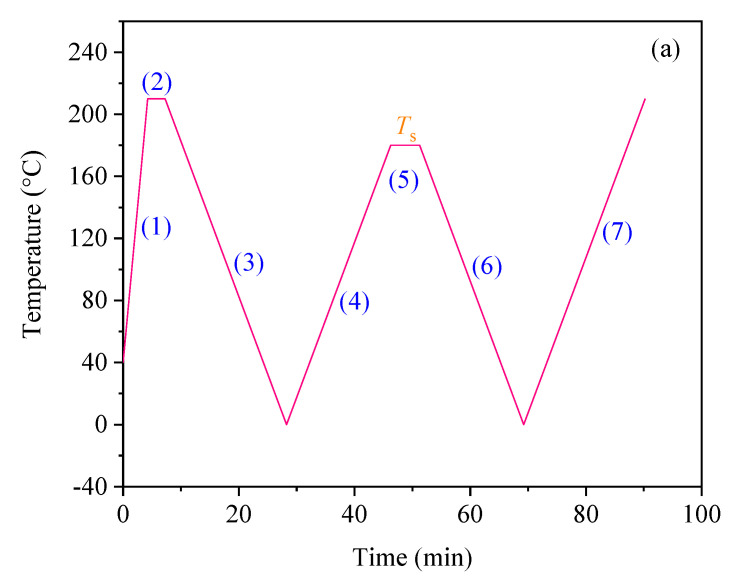

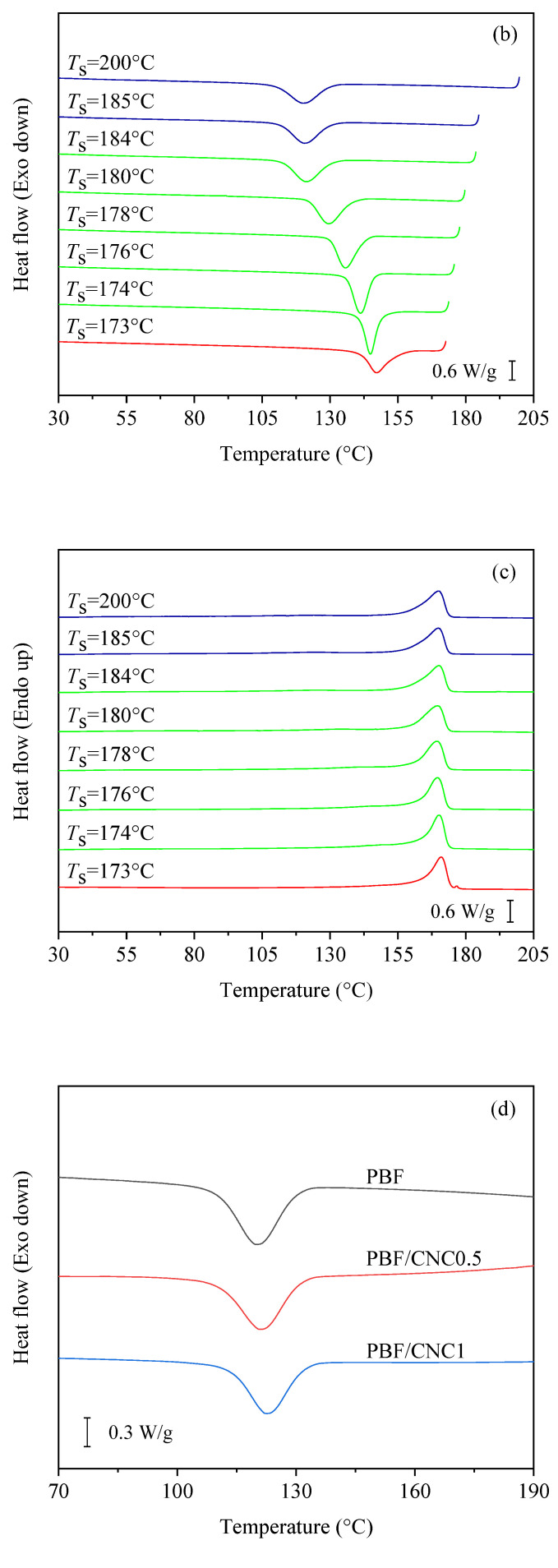
(**a**) Thermal procedure for the self-nucleation study of PBF, (**b**) DSC cooling traces of PBF from the indicated *T*_s_ at 10 °C/min (step 6), (**c**) DSC heating curves of PBF at 10 °C/min (step 7), and (**d**) DSC cooling curves at 10 °C/min.

To determine the *T*_cs_ value of PBF, a standard self-nucleation study was performed. Figure 4a shows the specific thermal procedure. Figure 4b,c depict the DSC traces of PBF after annealing at selected self-nucleation temperatures (*T*_s_) for 5 min. At *T*_s_ ≥ 185 °C, *T*_cc_ basically remained unchanged at approximately 120 °C, suggesting that the thermal history of PBF was completely eliminated; therefore, the nucleation of PBF occurred from the nuclei-free melt. At 174 °C ≤ *T*_s_ ≤ 184 °C, *T*_cc_ gradually shifted to a higher temperature (121.2 °C~144.8 °C), indicating that the self-nucleation of PBF occurred in the presence of residual nuclei or crystal fragments [47]. Two cases may occur within this *T*_s_ range. When *T*_s_ was close to 184 °C, the crystals were completely melted; however, certain melt memory, such as some residual chain orientation, may be retained in the melt, acting as the self-nuclei during the subsequent cooling process. In addition, when *T*_s_ was near 174 °C, most of the polymer crystals were molten; nevertheless, some crystal fragments still remained, acting as self-seeds in the subsequent cooling process. Consequently, *T*_cc_ significantly increased due to the presence of self-nuclei or self-seeds [47]. When *T*_s_ continually dropped to 173 °C, *T*_cc_ further increased to 147.1 °C; moreover, in addition to the main melting peak near 170 °C, a small new melting peak appeared at around 176.7 °C, as depicted in Figure 4c, indicating that both self-nucleation and annealing occurred simultaneously. The *T*_cs_ of PBF was thus determined to be 144.8 °C. Table S2 summarizes the relevant data from the self-nucleation study.

Figure 4d displays the DSC cooling curves of PBF and PBF/CNC composites at 10 °C/min. As seen in Figure 4d, the *T*_cc_ of PBF was 120.0 °C, while the *T*_cn_ values of PBF/CNC0.5 and PBF/CNC1 were 121.1 and 122.6 °C, respectively. Through Equation (4), the *NE* value of PBF/CNC0.5 was 4.4%, while that of PBF/CNC1 was 10.5%. In our previous study, the *NE* of PHF/CNC composites with the same content of CNC were 28.6% and 42.9%, respectively, indicating again that CNC as a nucleating agent was more effective in PHF than in PBF with the same content.

### 3.2. Isothermal Melt Crystallization Kinetics and Crystal Structure Study

In the above section, the low loadings of CNC were found to be able to promote the nonisothermal melt crystallization behavior of PBF; moreover, both the nucleation activity and the nucleation efficiency of CNC on the crystallization of PBF were quantitively studied. In this section, the influence of CNC on the isothermal melt crystallization kinetics of PBF was studied at different crystallization temperatures.

**Figure 5:**
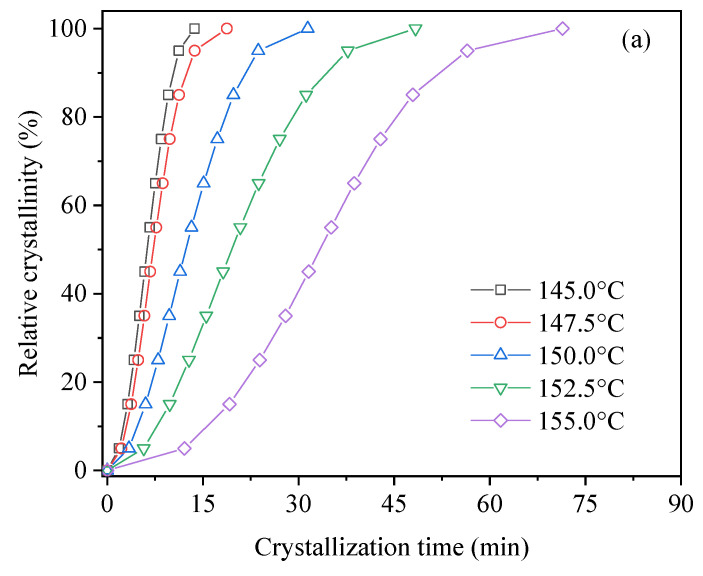

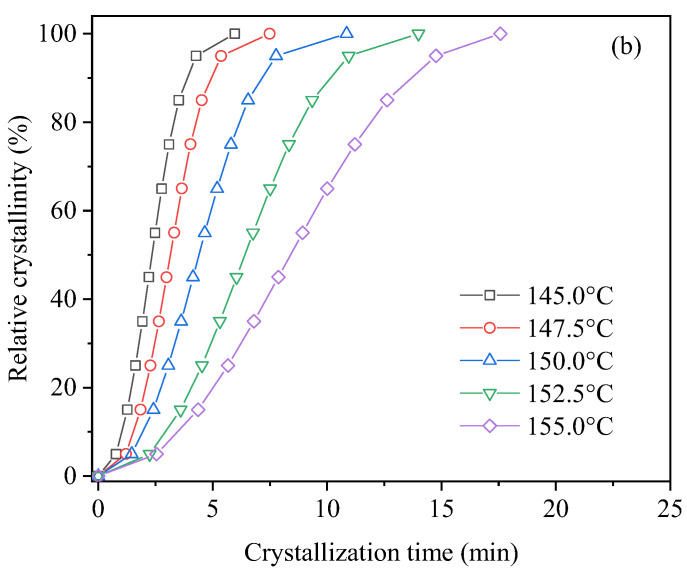
Plots of relative crystallinity versus crystallization time of (**a**) PBF and (**b**) PBF/CNC1.

Figure 5 depicts the variations in relative crystallinity with crystallization time for PBF and PBF/CNC1 at selected crystallization temperature (*T*_c_) values. With increasing *T*_c_, the crystallization time obviously became longer for both PBF and its composite. For instance, the crystallization time significantly increased from 13.7 to 71.4 min with increasing *T*_c_ from 145 to 155 °C for PBF, indicating a reduction in crystallization rate. The slowdown of the crystallization rate should be directly related to the decreased degree of supercooling. However, at the same *T*_c_, CNC remarkably shortened the crystallization time of PBF. For instance, at the same *T*_c_ of 155 °C, the crystallization time of PBF remarkably decreased from 71.4 to 17.6 min in the presence of only 1 wt% of CNC. Such a result indicated the role of CNC as an efficient biobased nucleating agent. Figure S2 shows the results for PBF/CNC0.5, displaying the similar effects of *T*_c_ and CNC to those described above.

**Figure 6:**
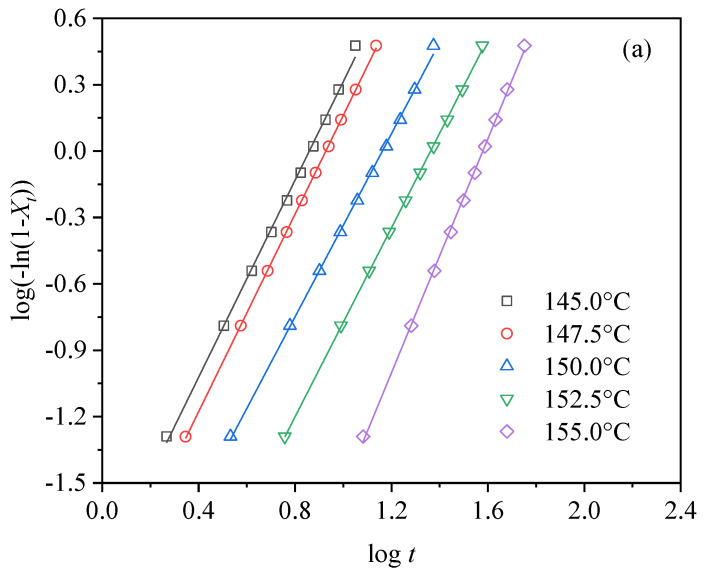

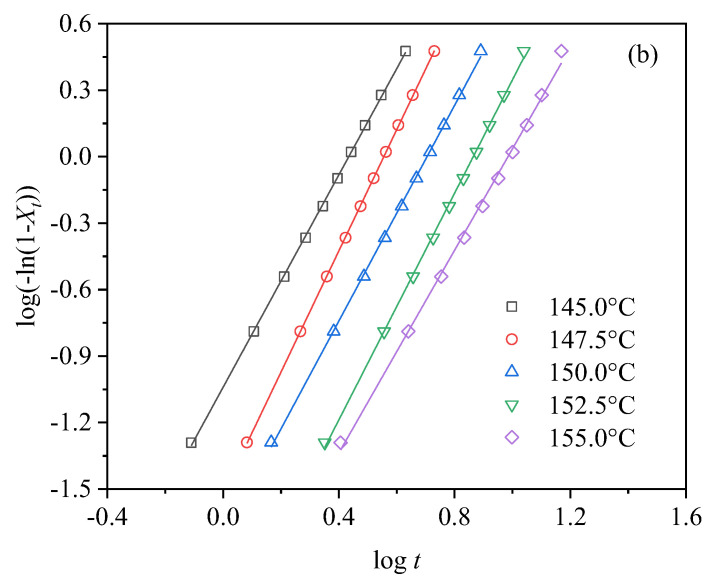
Avrami plots of (**a**) PBF and (**b**) PBF/CNC1.

The well-known Avrami equation was used to analyze the isothermal melt crystallization kinetics of PBF and PBF/CNC composites:

1 − *X*_t_ = exp(−*kt^n^*) (5)

where *X*_t_ is the relative crystallinity at crystallization time (*t*), *n* is the Avrami exponent, and *k* is the crystallization rate constant [48,49]. Figure 6 shows the Avrami plots of PBF and PBF/CNC1. For both samples, five nearly parallel fitting lines were observed, suggesting that the Avrami equation was suitable to describe the isothermal crystallization kinetics. Figure S3 illustrates the Avrami plots for PBF/CNC0.5, demonstrating the similar results shown in Figure 6.

From the Avrami plots, the relevant kinetic parameters were obtained. The relevant Avrami parameters were summarized in Table 1 for both PBF and PBF/CNC composites. As seen in Table 1, *n* slightly varied from 2.1 to 2.7 within the studied *T*_c_ range, confirming that the crystallization mechanism of PBF was not modified by CNC. By utilizing crystallization half-time (*t*_0.5_), crystallization rates were directly compared, which were derived through the following equation:

(6) $$t_{0.5}={\left(\frac{\ln2}{k}\right)}^{1/n}$$

The obtained *t*_0.5_ values are also summarized in Table 1.

**Table 1:** Kinetics parameters of PBF and PBF/CNC composites during isothermal melt crystallization.

Samples	*T*_c_ (°C)	*n*	*k* (min^−^*^n^*)	*t*_0.5_ (min)
PBF	145.0	2.2	1.22 × 10^−2^	6.1
	147.5	2.2	8.49 × 10^−3^	7.4
	150.0	2.1	3.94 × 10^−3^	12.2
	152.5	2.1	1.23 × 10^−3^	19.4
	155.0	2.6	6.67 × 10^−5^	33.0
PBF/CNC0.5	145.0	2.3	4.72 × 10^−2^	3.2
	147.5	2.6	8.16 × 10^−3^	5.5
	150.0	2.4	6.18 × 10^−3^	7.3
	152.5	2.5	1.52 × 10^−3^	12.0
	155.0	2.4	1.04 × 10^−3^	15.0
PBF/CNC1	145.0	2.4	9.11 × 10^−2^	2.3
	147.5	2.7	3.02 × 10^−2^	3.1
	150.0	2.4	1.93 × 10^−2^	4.4
	152.5	2.6	6.15 × 10^−3^	6.2
	155.0	2.3	5.59 × 10^−3^	8.1

**Figure 7:**
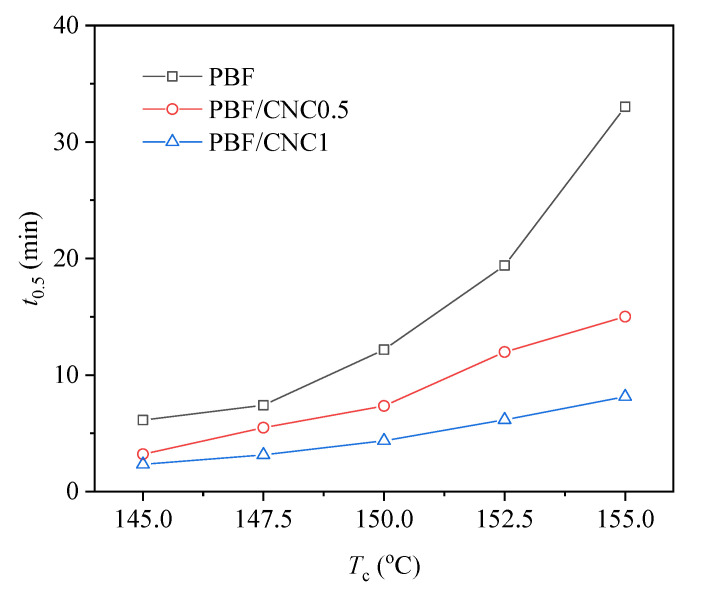
Plots of *t*_0.5_ versus *T*_c_ for PBF and PBF/CNC composites.

To clearly show the influence of *T*_c_ and CNC loading on the crystallization rate of PBF, Figure 7 demonstrates the plots of *t*_0.5_ versus *T*_c_ for PBF and PBF/CNC composites. As shown in Figure 7, the *t*_0.5_ of each sample gradually decreased as the *T*_c_ decreased, indicating a faster crystallization rate because of the increased degree of supercooling. In addition, at the same *T*_c_, the composites displayed obviously smaller *t*_0.5_ values than PBF; furthermore, the *t*_0.5_ values gradually decreased with increasing CNC loading. Such results suggested that CNC obviously accelerated the isothermal melt crystallization of PBF as a biobased nucleating agent.

In brief, the above study confirmed the nucleation agent role of biobased CNC under different crystallization processes. For instance, in the presence of only 1 wt% of CNC, the *T*_p_ of PBF increased from 126.6 to 130.0 °C at the same cooling rate of 5 °C/min, while the *t*_0.5_ of PBF remarkably decreased from 33.0 to 8.1 min at the same *T*_c_ of 155 °C. Herein, the possible nucleation mechanism was further discussed. Under different crystallization conditions, CNC provided additional foreign surfaces; consequently, the PBF chain was easily attached and folded on the surface. The heterogeneous nucleation of PBF became stronger in the presence of CNC, thus promoting the crystallization process and increasing the crystallization rate of PBF.

**Figure 8:**
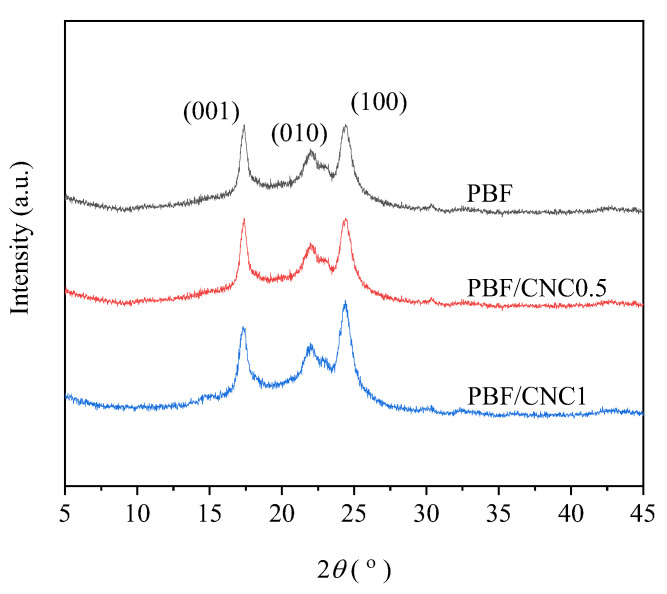
WAXD profiles for PBF and PBF/CNC composites.

The crystal structures of PBF and PBF/CNC composites were further studied through wide-angle X-ray diffraction (WAXD), after first crystallizing at 150 °C for 6 h from the molten state in an oven. As illustrated in Figure 8, both PBF and PBF/CNC composites demonstrated three strong diffraction peaks at around 2*θ* = 17.4°, 22.0°, and 24.4°, which were attributed to the (001), (010), and (100) crystal planes of PBF, respectively [15]. Consequently, despite the presence of CNC, the crystal structure of the PBF matrix remained unmodified.

### 3.3. Enhanced Mechanical Property and Hydrophilicity of PBF by CNC

**Figure 9:**
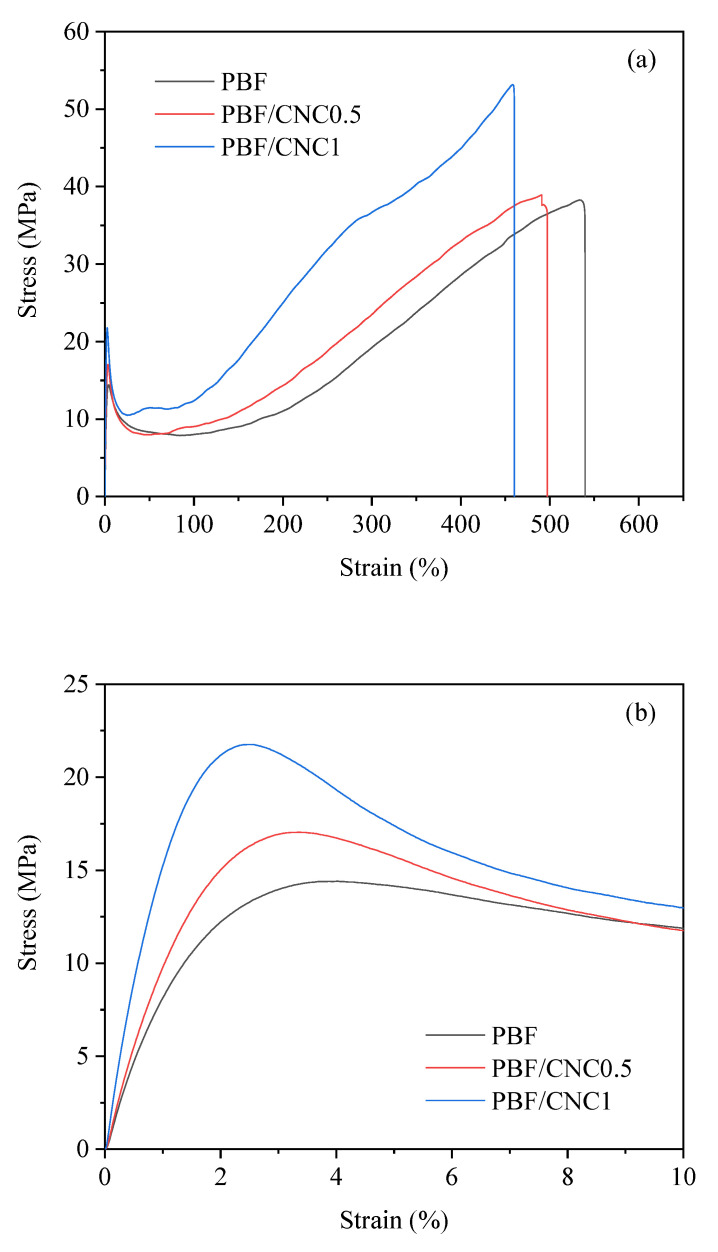
(**a**) Stress–strain curves and (**b**) enlarged stress–strain curves at low strain of PBF and PBF/CNC composites.

In this section, the mechanical properties and hydrophilicity of PBF and PBF/CNC composites were further investigated to explore the influence of CNC. Figure 9a,b illustrate the overall and partially magnified stress–strain curves of PBF and its composites, respectively. As seen in Figure 9a, all samples displayed plastic deformation and obvious yielding behaviors. Table 2 shows the mechanical property data. With increasing CNC loading, there was a remarkable increase in the Young’s modulus (*E*_t_) from 107.2 ± 5.9 MPa for PBF to 131.4 ± 7.3 MPa for PBF/CNC0.5 and 186.5 ± 8.6 MPa for PBF/CNC1, respectively. The tensile strength (*σ*) also significantly increased from 35.4 ± 2.9 MPa for PBF to 40.8 ± 2.1 and 50.7 ± 3.0 MPa for the two composites, respectively. The significant improvement in *E*_t_ and *σ* should be related to the following two factors. On the one hand, CNC with rigidity and high strength acted as a biobased reinforcing filler in the PBF matrix [32]. On the other hand, the crystallinity of PBF increased in the presence of CNC, resulting in the mechanical enhancement of the composites [50]. In our previous study, the yield strength and the Young’s modulus of PHF/CNC composites were also improved, indicating the good mechanical enhancement effect of CNC by both PBF and PHF. The elongation at break (*ε*) only decreased gradually to 494.5 ± 3.5% and 467.1 ± 7.2% for PBF/CNC0.5 and PBF/CNC1, respectively, from 545.0 ± 5.9% for PBF. In short, the low loadings of CNC obviously reinforced PBF without greatly sacrificing the toughness of PBF. The improved mechanical property of PBF by the low loadings of CNC should be essential from an application viewpoint, especially as packaging material.

**Figure 10:**
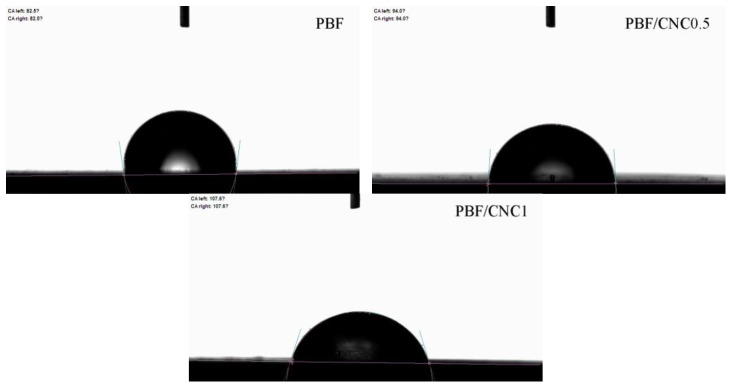
Water contact angle measurement of PBF and PBF/CNC composites.

The influence of CNC on the hydrophilicity of PBF was further studied. Figure 10 depicts the water contact angle (WCA) images of PBF and PBF/CNC composites. PBF was clearly a hydrophobic material with a WCA value of 97.1 ± 0.7°, while PBF/CNC0.5 and PBF/CNC1 exhibited increased hydrophilicity with the WCA values of 85.4 ± 0.6° and 72.8 ± 0.4°, respectively. The deposition of CNC on the surface of the film would significantly lead to a change in the water contact angle; therefore, the dispersion of CNC was first observed with a scanning electron microscope. From Figure S4, CNC uniformly dispersed in the PBF matrix of the studied CNC contents. Therefore, the decrease in WCA value should be related to the large amount of hydroxyl groups on the surface of CNC, making it strongly hydrophilic and thereby enhancing the hydrophilicity of PBF. The increase in hydrophilicity may accelerate the hydrolysis of PBF and influence other physical properties, which is still underway and will be reported in the forthcoming research.

## 4. Conclusions

The low loadings of CNC on nucleated and reinforced biobased PBF composites were successfully prepared in this research through a solution and casting method. The influence of the low loadings of CNC on the crystallization behavior, mechanical property, and hydrophilicity of PBF was systematically studied. Due to the occurrence of the secondary crystallization, the Ozawa equation was not suitable to fit the nonisothermal melt crystallization kinetics. Both the nucleation activity and the nucleation efficiency of CNC were quantitatively evaluated in the PBF matrix. They were 0.98 and 4.4% at 0.5 wt% of CNC, while they were 0.94 and 10.5% at 1 wt% of CNC, suggesting an increased nucleation effect. During different crystallization conditions, CNC accelerated the crystallization of PBF as a biobased nucleating agent. For instance, the *T*_p_ of PBF increased from 126.6 to 130.0 °C during the nonisothermal melt crystallization at 5 °C/min, while the *t*_0.5_ of PBF remarkably decreased from 33.0 to 8.1 min during the isothermal melt crystallization at 155 °C by the presence of only 1 wt% of CNC. In the fully biobased composites, CNC did not modify the crystallization mechanism and the crystal structure of PBF as a biobased nucleation agent; however, CNC remarkably enhanced the mechanical properties of PBF as a rigid reinforcing filler. Only 1 wt% of CNC significantly increased the Young’s modulus and the tensile strength of PBF from 107.2 ± 5.9 and 35.4 ± 2.9 MPa to 186.5 ± 8.6 and 50.7 ± 3.0 MPa, respectively. The hydrophilicity of PBF also increased in the composites. For instance, the WCA value of PBF obviously decreased from 97.1 ± 0.7° to 72.8 ± 0.4° by the presence of 1 wt% of CNC due to the large amount of hydroxyl groups on the surface. In sum, the low loadings of CNC simultaneously enhanced the crystallization, mechanical property, and hydrophilicity of PBF, which must be significantly important and interesting from a dual carbon strategy perspective.

## Figures and Tables

**Table 2:** Mechanical property data of PBF and PBF/CNC composites.

Samples	Young’s Modulus (MPa)	Tensile Strength (MPa)	Elongation at Break (%)
PBF	107.2 ± 5.9	35.4 ± 2.9	545.0 ± 5.9
PBF/CNC0.5	131.4 ± 7.3	40.8 ± 2.1	494.5 ± 3.5
PBF/CNC1	186.5 ± 8.6	50.7 ± 3.0	467.1 ± 7.2

## Data Availability

The original contributions presented in this study are included in the article/Supplementary Materials. Further inquiries can be directed to the corresponding author.

## Supplementary Materials

The following supporting information can be downloaded at: https://www.mdpi.com/article/10.3390/polym17020196/s1, Figure S1. Crystallization exotherms of (a) PBF, (b) PBF/CNC0.5, and (c) PBF/CNC1 at different cooling rates; Figure S2. Plots of relative crystallinity versus crystallization time of PBF/CNC0.5; Figure S3. Avrami plots of PBF/CNC0.5; Figure S4. SEM images of the fractured surfaces of (a) PBF, (b) PBF/CNC0.5, and (c) PBF/CNC1; Table S1. he relevant data during the nonisothermal melt crystallization process at different cooling rates; Table S2. The relevant data from the self-nucleation study.
