# Supplementary material for: Influence of Low Loadings of Cellulose Nanocrystals on the Simultaneously Enhanced Crystallization Rate, Mechanical Property, and Hydrophilicity of Biobased Poly(butylene 2,5-furandicarboxylate)
Source: Polymers (Basel). 2025 Jan 14;17(2):196. doi: 10.3390/polym17020196 (PMC11768259; doi:10.3390/polym17020196)
Supplement: Supplementary file 1 [file polymers-17-00196-s001.zip › polymers-3397444-supplementary.pdf]

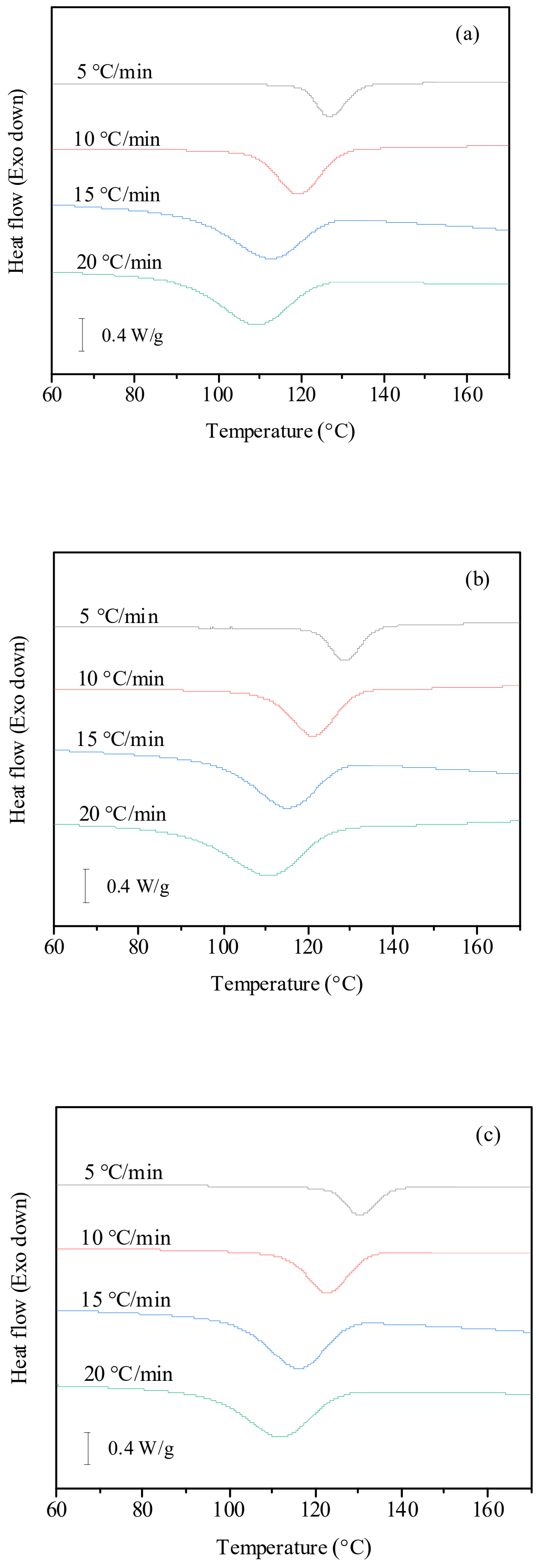

Figure S1. Crystallization exotherms of (a) PBF, (b) PBF/CNC0.5, and (c) PBF/CNC1 at different cooling rates.

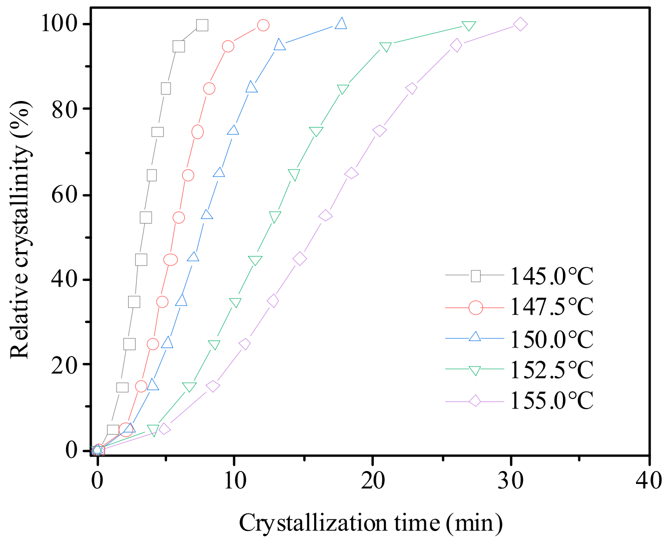

Figure S2. Plots of relative crystallinity versus crystallization time of PBF/CNC0.5.

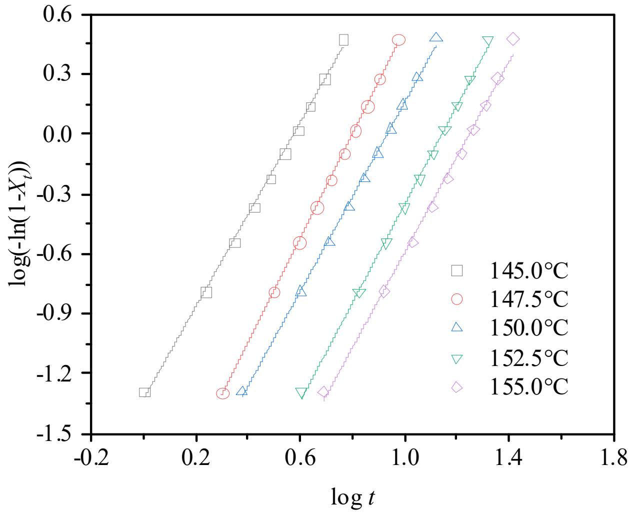

Figure S3. Avrami plots of PBF/CNC0.5.

Table S1. The relevant data during the nonisothermal melt crystallization process at different cooling rates.

Samples	Φ (°C/min)	T_P (°C)	ΔH_P (J/g)
PBF	5	126.6	48.8
	10	119.1	47.3
	15	111.8	42.3
	20	108.7	37.9
PBF/CNC0.5	5	128.5	51.1
	10	121.1	50.6
	15	114.6	44.0
	20	110.8	41.3
PBF/CNC1	5	130.0	51.4
	10	122.6	49.7
	15	115.7	44.5
	20	111.6	39.2

Table S2. The relevant data from the self-nucleation study.

T_s (°C)	T_{cc} (°C)	ΔH_{cc} (J/g)	T_m (°C)	ΔH_m (J/g)
200	120.0	48.3	169.8	48.9
185	120.3	47.5	169.9	48.3
184	121.2	48.6	170.1	49.0
180	129.5	47.9	169.6	48.7
178	135.7	50.2	169.4	50.5
176	141.2	50.8	169.6	51.2
174	144.8	49.1	170.1	51.6
173	147.1	48.8	170.9/176.7	49.7

The dispersion of CNC in the composites was observed by a scanning electron microscope (SEM, JEOL JSM-7800F). The observed surfaces were obtained after being fractured into liquid nitrogen.

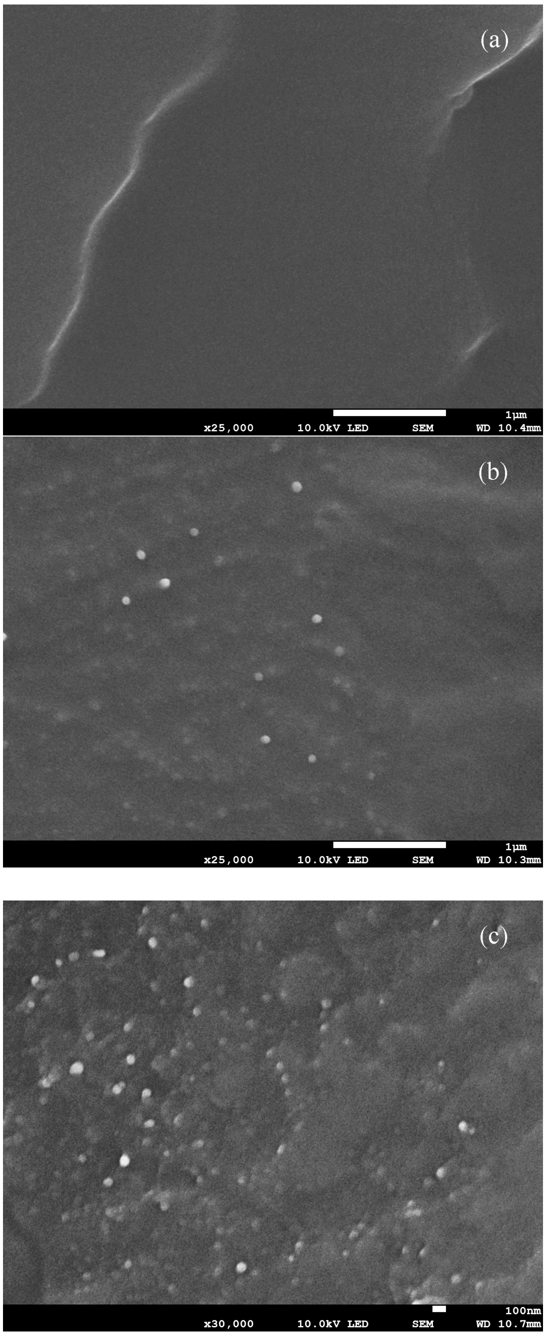

Figure S4. SEM images of the fractured surfaces of (a) PBF, (b) PBF/CNC0.5, and (c) PBF/CNC1.
